# Immunohistochemical Markers of Temporomandibular Disorders: A Review of the Literature

**DOI:** 10.3390/jcm12030789

**Published:** 2023-01-18

**Authors:** Luis Eduardo Almeida, Andrea Doetzer, Matthew L. Beck

**Affiliations:** 1 Surgical Sciences Department, School of Dentistry, Marquette University, Milwaukee, WI 53233, USA; 2 Faculdade de Odontologia, Pontificia Universidade Catolica do Parana, Curitiba 80215-901, Brazil

**Keywords:** temporomandibular joint, temporomandibular disorders, biomarkers, immunohistochemistry, systematic review

## Abstract

Temporomandibular disorders (TMD) are a group of internal derangements encompassing dysfunction, displacement, degeneration of the temporomandibular joints and surroundings muscles of mastication, often accompanied by pain. Relationships between TMD and various chemical biomarkers have been examined throughout the years. This paper aims to gather evidence from the literature regarding other biomarkers and presenting them as one systematic review to investigate the potential links between TMD and different biochemical activity. To identify relevant papers, a comprehensive literature search was carried out in MEDLINE/PubMED, EMBASE, Web of Science and a manual search was performed in the International Journal of Oral and Maxillofacial Surgery, Journal of Oral and Maxillofacial surgery, and Journal of Cranio-Maxillo-Facial Surgery. The literature review produced extensive results relating to the biochemical and immunohistochemical markers of TMD. Many enzymes, inflammatory markers, proteoglycans, and hormones were identified and organized in tables, along with a brief description, study design, and conclusion of each study. Through this review, recurring evidence provides confidence in suggesting involvement of certain biomarkers that may be involved in this complex pathogenesis, in addition to pointing to differences in gender prevalence of TMD. However, more organized research on large human samples needs to be conducted to delve deeper into the understanding of how this disease develops and progresses.

## 1. Introduction

The temporomandibular joint (TMJ) is a ginglymoarthrodial joint acting as a union of the mandibular fossa of the temporal bone and the head of the mandibular condyle. It is used in the mastication processes via translational (gliding) and rotational (hinging) movements. Its primary components include the articular disc, the articular capsule, innervation and vascularization, ligaments, and the muscles of mastication [1].

The inner region of the TMJ contains an articular disc composed of fibrous connective tissue devoid of any blood vessels and nerves. It is attached to the capsular ligament anteriorly, posteriorly, medially, and laterally to separate the TMJ into two cavities. Posteriorly, loose connective tissue is attached to the connective tissue and is highly vascularized and innervated. Superiorly, the fibroelastic fascia attaches with elastic fibers to the tympanic plate. Inferiorly, at the posterior edge of the disc is the inferior retrodiscal lamina composed of fibrous, collagenous fibers. The disc is attached to the condylar formation at the medial and lateral aspects. The remaining body of the retrodiscal tissue is attached posteriorly to a large venous plexus that fills with blood as the condyle moves forward.

The TMJ is surrounded by the articular capsule, a capsular ligament with specialized endothelial cells that form a synovial lining which provides boundary and weeping lubrication. These cells provide synovial fluid, which act as a medium to prevent friction in the moving joint (boundary lubrication), as well as the ability of the articular surface to absorb a small amount of the synovial fluid in a compressed or nonmoving joint to eliminate friction (weeping lubrication) [1].

The supportive ligaments of the TMJ are collagenous and do not stretch, acting passively to restrain and limit border movements. These ligaments include the collateral, the capsular, and the temporomandibular ligaments. The collateral ligament serves to anchor the condyle. It is used to restrict movement of the disc away from the condyle and permits the hinge movement of the condyle. The capsular ligament surrounds the TMJ and attaches the temporal bone to the neck of condyle, resisting medial, lateral, and inferior forces that may separate or dislocate the articular surfaces. The temporomandibular ligament is composed of two portions: an oblique portion the connects the articular tubercle to the outer portion of the neck and resists excessive dropping of the condyle, and the horizontal portion that connects the articular tubercle to the tip of the condyle, limiting posterior movement of the condyle and disc. The accessory ligaments of the TMJ include the sphenomandibular and the stylomandibular, which prevent excess protrusive movements [1].

Temporomandibular disorders (TMD) are a group of internal derangements encompassing dysfunction, displacement, degeneration of the temporomandibular joints and surrounding muscles of mastication, often accompanied by pain. Currently, TMD is classified based on the articular disc and its mobility during mandibular movements. In early disease, the disc is displaced anteriorly, but returns to its normal position upon opening—termed anterior disc displacement with reduction (ADDwR). In later disease, the disc remains displaced even upon mandibular movements, creating a limitation in mobility and causing increased pain—termed anterior disc displacement without reduction (ADDwoR). Eventually, osteoarthritic changes may occur. The classic method of differentiating the stage of the disease was created by Wilkes. In his description, Wilkes uses clinical, radiographic and surgical findings to categorized cases into early, early/intermediate, intermediate, intermediate/late, and late stages [2].

It has been suggested that up to 25% of the population may experience symptoms of TMD, with a gender bias existing in which women are more affected by a ratio suggested to be greater than 2:1 [3]. Symptoms typically include clicking and popping of the joint, usually in early stages, limited range of motion and function, and orofacial pain. Current treatment modalities range from non-invasive options, such as physical therapy and occlusal splints, to minimally invasive options, such as trigger point injections and arthrocentesis, to invasive therapies, such as total joint replacement.

Despite our current understanding of TMD, the exact etiology of the disease remains unknown. It is crucial we develop our understanding of the progression and complications in order to diagnose and treat the disorder more effectively.

The relationships between TMD and various chemical biomarkers have been examined throughout the years. Matrix metalloproteinases (MMPs) are the major enzymes involved in extracellular matrix and basement membrane remodeling. Furthermore, they play a role in regulating chemokines, growth factors, proteases, among other molecules, in order to balance the inflammatory response [4]. Interleukins (IL) are a group of proteins that regulate cell growth, differentiation and motility. There are fifteen different interleukins known, and many of these are important factors in the inflammatory immune response [5]. These markers have the potential to be diagnostic and therapeutic targets for treating and managing TMD. MMPs and ILs are just a few of the immunohistochemical markers associated with TMD, and this paper aims to gather evidence from the literature regarding other common markers and presenting them as one systematic review to investigate the potential links between TMD and different biochemical activity.

## 2. Materials and Methods

### 2.1. Search Protocol

The MEDLINE/PubMed, EMBASE, and Web of Science databases were searched between January 2020 and March 2020. The manual search was performed in the three main journals of the field (International Journal of Oral and Maxillofacial Surgery, Journal of Oral and Maxillofacial Surgery, and Journal of Cranio-Maxillo-Facial Surgery), as well as from the reference list of studies included in this systematic review.

For the construction of the search strategy, MeSH (Medical Subject Heading) terms were used, which are considered descriptors of controlled subjects for searching MEDLINE, Web of Science, and Cochrane. To make the search more sensitive, vocabularies not controlled by the use of keywords were included. Entree terms were used for the search strategy in EMBASE.

The search was carried out by combining the terms (“MeSH” and keywords) for population and intervention, with the help of the Boolean operators “OR” and “AND”. The strategy adopted for the MEDLINE/PubMed base was as follows: (TMJ OR temporomandibular joint OR TMD OR temporomandibular dysfunction OR temporomandibular joint dysfunction OR temporomandibular joint derangement) “AND” (immunohistochemistry OR immune antibody OR proteomic OR protein expression). In addition to these terms, the following keywords were used: disc displacement OR TMJ internal derangement OR TMD. The PRISMA 2020 statement was used as reference in reporting this systematic review. (Page, M.J.; McKenzie, J.: Bossuyt, P.: Boutron, I.; Hoffman, T.; Mulrow, C.; Shamseer, L.; Tetzlaff, J.; Akl, E.; Brennan, S.E.; et al. The Prisma 2020 Statement: An Updated Guideline for reporting Systematic Reviews. Syst. Rev. 2020, 10, 89.) The inclusion criteria was: human and animal research with a definitive clinical diagnosis or specific signs and symptoms for TMD, and protein expression results obtained through either immunohistochemistry OR immune antibody OR proteomic OR protein expression. The exclusion criteria were as follows: studies not in English, full text not available, studies not related to TMDs or where there was no mention of specific diagnoses or signs and symptoms, studies without the use of control to compare sample with and without protein expression.

**Figure 1:**
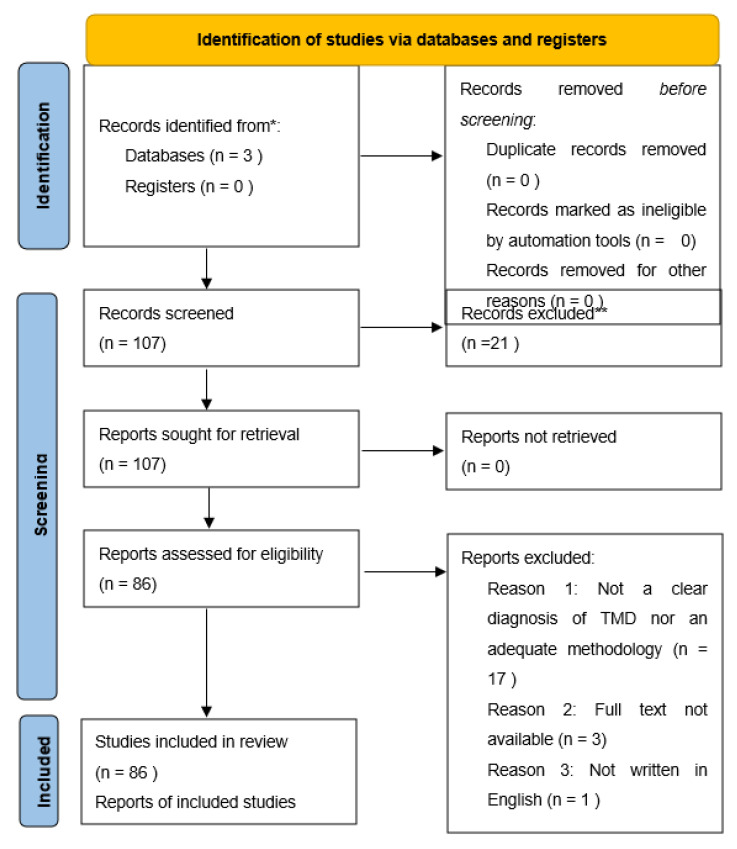
PRISMA 2020 flow diagram. * MEDLINE, Web of Science, and Cochrane, ** EMBASE.

A total of 107 studies were analyzed but only 86 were in accordance with PRISMA’s criteria. The excluded studies either did not have a clear diagnosis of TMD or an adequate methodology. Three studies did not have the full text available, and one was not written in English. (Figure 1).

### 2.2. Data Analysis

The search protocol that was used was designed to be as inclusive as possible to encompass as many potential biomarkers that may be involved in the pathogenesis of TMD. As such, the methodology of the included studies varies widely, from utilization of different detection techniques in analyzing biomarker involvement, such as polymerase chain reaction (PCR), DNA extraction, immunohistochemical staining (IHC), among many others, to subject species—such as detection of biomarkers in human, rabbit or mouse tissues. With such variation in study design, a direct analysis of the data obtained through the included studies was unable to be conducted. Furthermore, the quality of each study, such as looking at statistical power, was not considered beyond the impact of the study based on factors such as sample size and the species from which the tissue was harvested in the study.

## 3. Results

The literature review produced extensive results relating to the biochemical and immunohistochemical markers of TMD. The most studied are: enzymes, such as matrix metalloproteinases (MMP), aggrecanase, and cyclooxygenase (COX); cytokines and inflammatory markers, such as interleukins (IL), tumor necrosis factor- alpha (TNF-alpha), and vascular endothelial growth factor (VEGF); proteoglycans, such as tenascin, vimentin and fibronectin; hormones, such as estrogen, progesterone and relaxin; and miscellaneous markers that did not fit into the other categories, such as aquaporin, heat shock protein, and b-cell lymphoma-2 gene (BCL-2). Each study, along with a brief description, study design, and the conclusions, are organized in the tables below.

## 4. Discussion

### 4.1. Enzymes

Matrix metalloproteinases (MMPs) have been a large target of research attempts to understand the underlying pathogenesis of TMD. As previously mentioned, MMPs are a family of 26 proteins that are involved in extracellular matrix and basement membrane remodeling through the breakdown of collagen, gelatin, proteoglycans, and other components of the basement membrane. One of the first studies looking at MMPs in the discs of the TMJ was conducted by Kapila, using various methods, such as Western blotting and immunohistochemical staining, to identify the expression of MMPs (gelatinases, collagenases and stromelysin) in the discs of rabbit TMJ [4]. Many studies followed and showed the strongest evidence for the involvement of MMP-2 and MMP-9, with some evidence showing the involvement of MMP-7 (Kapila, [5], Tanaka, [6], Yoshida, [7], Loreto, [8,9], Almeida et al. [10]). Perroto [11], however, did not identify a significant difference in levels of MMP-13 between samples of human discs with TMD when compared to control samples. This points to the complex nature of basement-membrane-remodeling regulation involved in TMD. Evidence suggests that MMP-2 and MMP-9 are involved in the pathogenesis of TMD; however, more research needs to be conducted to support the involvement of the other suggested MMPs (MMP-1, MMP-7) and to definitively exclude those that have not been shown to be involved thus far (MMP-13). (Table 1).

### 4.2. Cytokines and Inflammatory Markers

Our review of the literature found several different cytokines and inflammatory markers to be in association with TMD, including interleukins, CD antigens, TNF alpha, TGF beta, VEGF, IGF, NFkB, IFN-y, capsaicin, and bradykinin. These markers are involved in many pathophysiological functions, including initiating and mediating inflammatory responses, activating immune cells, regulating apoptosis and angiogenesis, and regulating pain and fever.

As previously mentioned, the interleukins are a major family of cytokines that are extensively involved in the immune response. They are secreted by macrophages and T cells in order to modulate inflammation and fever, activate and maintain immune cells and bone marrow, produce immunoglobulins and acute phase proteins, and maintain granulomas. Various studies in our review found IL-1, IL-2, IL-6, IL-8, and IL-10 to be associated with TMD. Kim (2012) did not detect IL-4 and IL-5 in either the TMD group or the control group during a study of human synovial fluid [23]. Fu et al. (1995), Ogura et al. (2002), and Sato et al. (2003) found an association between IL-6 and TMD, while Caporal et al. (2017) found no significant difference when comparing TMD groups of ADDwR and ADDwoR, and with and without OA [24,25,26,27]. (Table 2).

### 4.3. Proteoglycans

The review of the literature found several different proteoglycans that are involved with TMD, such as tenascin, vimentin, fibronectin, decorin, biglycan, veriscan, and elastin. Proteoglycans are proteins located in the extracellular matrix, cell surface, or intracellular granules. They are involved in several processes, such as cell signaling and organization of the extracellular matrix [53]. Yoshida and Leonardi found that tenascin was associated with degenerated tissue, specifically in the portion of the TMJ synovial membrane affected by internal derangement [54]. Additionally, Toriva et al. (2006) [55] found that versican is associated with TMD and causes growth-related changes and regional differences in the TMJ discs of rats. The full table of proteoglycans and their respective results may be seen in the table above. (Table 3).

### 4.4. Hormones

Our search of the literature resulted in seven studies looking at the involvement of three hormones in the pathogenesis of TMD. These studies examined relaxin, estrogen and progesterone in tissues from rabbit, mice and baboon discal tissues. Relaxin is a hormone that has been shown to be involved in matrix remodeling during pregnancy, and its effects are potentiated in the presence of estradiol [75]. A common finding among the studies conducted by Naqvi, Kapila, Hashem, and Park showed that an increased level of relaxin in disc samples was associated with increased levels of MMPs, such as collagenase and stromelysis, and a decrease in the levels of collagen and glycosaminoglycans (GAGs) [4,71,75]. Hashem, however, also found that the hormone progesterone prevented the effects of relaxin and estrogen, limiting the amount of matrix loss by preventing induction of MMPs [76].

An alternative methodology of looking at the hormonal involvement in the pathogenesis was utilized in some studies by examining the estrogen and/or relaxin receptor. Wang’s study examined the level of estrogen receptors α and β, relaxin receptors LGR7 and LGR8, and progesterone receptor in various joints, and they found a higher number of hormone receptors in the TMJ of mice when compared to the knee joint [76]. In a study conducted by Puri, it was found that the level of estrogen-α receptors in the TMJ, however, could be influenced by inflammation—an increase in inflammation was correlated with a decrease in estrogen-α receptors [77]. Together, these help illustrate the multifactorial etiology that makes understanding this disorder so difficult.

These findings support the idea that hormonal influences are a key factor in the gender disparity associated with TMD. With studies showing a large number of hormonal receptors located in the joint, and with studies showing an increase in relaxin and estrogen associated with increased MMP activity and decreased collagen and GAGs, evidence points toward hormones playing a key role in this disparity. Although these studies suggest this, however, it is important to note that all studies were carried out on different species, and more research should be conducted with human samples for stronger support. Similarly, future studies should focus on specific receptors and hormones to identify which hormones have the largest impact on the pathogenesis, as well as look at the role of inflammation. (Table 4).

### 4.5. Miscellaneous

Many of the studies that resulted from our search of the current literature included biomarkers that do not fit the preceding categories. Leonardi et. al. looked at the levels of heat shock protein 27 (HSP-27) in human disc tissues and found that an increased level of the protein was associated with TMD, while the level of HSP-27 was virtually undetectable in samples from fetal tissue and normal joint tissue [79]. Heat shock proteins act within cells to protect against protein denaturation; however, they have been suggested to be involved in the pathogenesis of many diseases with elevated levels for prolonged periods of time.

Huang et al. looked at the presence of BCL-2 and BAX in disc tissue from rabbit samples [80]. BCL-2 is a protooncogene that is anti-apoptotic in nature, while BAX is a pro-apoptotic gene. Immunohistochemical staining of the disc samples revealed the presence of receptors for both BCL-2 and BAX, which suggests the possibility of their involvement in the regulation of the apoptotic process of TMD [80]. With respect to tumor suppressors, Castorina et. al. looked at the presence of P53 expression (along with VEGF) in human disc samples from subjects with TMD and found a positive correlation between pathological changes of the tissue and expression of the tumor suppressor protein P53, as well as levels of VEGF [81]. These studies are added evidence of the complex nature of the regulation of the apoptotic pathway in TMD.

Aquaporins are membrane channel proteins that allow water to pass through cell membranes to help regulate osmotic hemostasis [82]. They are found as part of normal physiology, with their presence identified in processes in the kidney, gastrointestinal tract, among other tissues, but have also been identified as part of various pathologic processes. In two studies carried out by Loreto, human disc samples were utilized to examine the presence of aquaporin-1 channels [53,83]. In the first study, it was found that these channels are part of normal homeostatic regulation of the TMJ; however, in the second study, it was found that the channel protein was upregulated in disc samples from subjects with TMD when compared with samples without TMD [82,84].

It has been previously shown that angiogenesis plays an important role in the progression of TMD, and, with that, the involvement of VEGF. Dickkopf-related protein (DKK-1) is a protein that inhibits the Wnt/beta-cantenin pathway and has been previously shown to play a role in physiologic and pathologic processes thorugh its role in promoting angiogenesis and recruitment of endothelial cells [85]. In a study conducted by Jiang, human synovial fibroblasts were examined for the presence of DKK-1 and found a high level of expression in the synovial fluid in samples obtained from subjects with TMD [84].

Elastin-derived proteins (EDPs) are bioactive peptides that arise with the degradation of elastin, a major component of the extracellular matrix [86]. An increase in elastin degradation and turnover has been shown to be a part of many pathological processes and through induction of inflammatory markers. Kobayachi used immunohistochemical staining to examine levels of EDPs in human synovial fluid and found an association between EDP, IL-6 and MMP-12, suggesting a role in EDPs in regulation of the inflammatory cascade in the TMJ [86].

Finally, in a study conducted by Fujimura, human synovial fluid was analyzed using electrophoresis in an attempt to look at synovial protein patterns [87]. The group found a significantly higher concentration of total protein in the synovial fluid of samples of patients with various stages of TMD when compared to healthy samples, especially in the amount of proteins with a higher molecular weight. Even when comparing the differences of protein concentration within the stages, a more advanced (osteoarthritis) stages were associated with a higher concentration of protein than earlier stages. These results support the idea that there is an increase in the level of inflammation as TMD progresses to higher stages. (Table 5).

## 5. Conclusions

The pathogenesis underlying the development and progression temporomandibular joint disorder is a very complex biochemical process. As evidenced by this review, much research has been conducted to identify the different underlying involvement of biological markers. Through this review, recurring evidence provides confidence in suggesting the involvement of matrix metalloproteinases that may be involved in basement membrane and disc pathogenesis, inflammatory markers that may be modulating pain and TMJ tissue breakdown, as well as point to differences in gender prevalence of TMD. Moreover, proteoglycans and several proteins were found to be involved in the inflammatory and apoptotic cascade contributing to TMD progression and severity. Evidence in the current literature has allowed us to increase the comprehension of the underlying pathophysiology of TMD; however, as mentioned throughout this paper, more organized research on larger human samples needs to be conducted in order to delve deeper into the understanding of how this disease develops and progresses.

## Figures and Tables

**Table 1:** Seven Enzymes (MMPs, aggrecanase, cyclooxygenase).

Authors	IHC Marker	Study Design and Tissue Expression	Results	Conclusions
Kapila et al. (1995) [12]	MMP	Rabbit discs	MMPs, namely proMMP-9, proMMP-2, proMMP-1, and proMMP-3 were detected in TMJ articular discs.	Proteinases characterized in rabbit TMJ discs
Marchetti et al. (1999) [13]	MMP-2	Human discs	Positive immunoreaction pattern for MMP-2. Fibroblast-, chondroblast- and osteoblast-like cells displayed a positive cytoplasmic reaction. Structural modifications of the articular disc could be specific responses to changes in the function of the TMJ.	MMP-2 produced disc alteration
Quinn et al. (2000) [14]	Cyclooxygenase-2	Human synovial fluid	COX-2 is present in the TMJ synovial tissue and fluid from patients with internal derangement. Therefore, COX-2 antagonists may be indicated in the treatment of TMJ arthralgia.	COX-2 expression in internal derangements
Tanaka et al. (2001) [6]	MMPs	Human synovial fluid	Quantitative analysis showed that the degree of MMP-2 and MMP-9 expression was higher in human patients with ADDwoR than in human patients with ADDwR.	MMP-2 and MMP-9 associated with TMD
Yoshida et al. (2001) [7]	Cyclooxygenase-1,2	Human synovial membranes	COX-1 may be an important mechanism for maintaining normal homeostasis at the endothelial cells and fibroblasts with internal derangement of TMJ.	Cyclooxygenase-1 associated with TMJ homeostasis
Puzas et al. (2001) [15]	MMPs and Cytokines	Mice discs	The presence of a MMP-9 (92-kD gelatinase) in TMJ disc and articular cells likely function in the degradative process. Additionally, this enzyme is under the control of pro-inflammatory cytokines whereby TGFbeta and IL-1 stimulate and PGE(2) inhibits its activity.	MMP-9 associated with degenerative processes
Yoshida et al. (2002) [16]	Cyclooxygenase-2	Human disk and synovial membranes	There were obvious distinction of COX-2 immunoreactivity between the control specimens and internal derangement cases, in the region of posterior and/or anterior loose connective tissues. intensive COX-2 expression was detected in the synovial membrane of internal derangement cases.	COX-2 expression and TMD
Yoshida et al. (2006) [17]	MMP and aggrecanase	Human synovial fluid	MMP-2, -9, and aggrecanase expression in the ID group were significantly higher than those in the normal group. Those with anterior disc displacement without reduction and severe OA showed significantly high expression of MMP-9 compared with other disease subgroups.	MMP-2, 9 and aggrecanase in TMD.
Yoshida et al. (2005) [18]	Aggrecanase	Human synovial fluid	Aggrecanase expression in TMJD group were significantly higher than that in the normal control group.	Aggrecanase associated with TMD
Hu et al. (2008) [8]	Urokinase-Type Plasminogen Activator and Urokinase-Type Plasminogen Activator Receptor	Human synovial fluid	uPA and uPAR in the synovial fluid may play a role in the pathogenesis of TMD, and the level of uPA and uPAR in synovial fluid of TMD could be used as a biochemical markers to reflect pathological degree of TMD.	uPA and uPAR associated with TMD
Matsumoto et al. (2008) [9]	Disintegrin and metalloproteinase with thrombospondin motifs 5 (ADAMTS-5)	Human discs	ADAMTS-5 is related to deformation and destruction of human TMJ discs affected by internal derangement.	ADAMTS-5 associated with TMD
Leonardi et al. (2010) [19]	Caspase 3	Human discs	Fatty degeneration is limited by apoptosis, with adipocytes being immunolabeled by caspase 3 antibody.	
Loreto et al. (2011) [20]	Caspase 3	Human discs	A greater proportion of caspase 3-positive cells were found in ADDwR and ADDwoR than in control discs.	Caspase 3 associated with TMD
Loreto et al. (2013) [11]	MMP-7 and MMP-9	Human discs	MMP upregulation in discs from patients contributes to disc damage.	MMP associated with TMD
Nascimento et al. (2013) [21]	MMP-2 and MMP-9	Rat trigeminal ganglion	MMP expression in the trigeminal ganglion was shown to vary during the phases of the inflammatory process. MMP-9 regulated the early phase and MMP-2 participated in the late phase of this process. Furthermore, increases in plasma extravasation in periarticular tissue and myeloperoxidase activity in the joint tissue, which occurred throughout the inflammation process, were diminished by treatment with DOX, a nonspecific MMP inhibitor.	MMP-2 and MMP-9 associated with process of TMJ inflammation
Almeida et al. (2015) [10]	MMP-2 and MMP-9	Human discs	MMP-2 expression was elevated in the disks of patients with displacement and without reduction. MMP-9 expression was not statistically elevated in the disks of patients with displacement and without reduction.	Elevation of MMP-2 in TMD.
Perroto et al. (2018) [22]	MMP-13	Human discs	MMP-13 is not significantly involved in collagen degradation.	Inflammatory cascade?

**Table 2:** Cytokines and Inflammatory Markers (Interleukins, TNF alpha, FasL, VEGF, IGF, NFKB, IFN-y, capsaicin, BMP-2, bradykinin).

Authors	IHC Marker	Study Design and Tissue Expression	Results	Conclusions
Fu et al. (1995) [24]	IL-6	Human synovial fluid	Interleukin-6 level was greater than 100 U/mL in 13 of 18 patients with degenerative joint disease and in 5 of 12 patients with disc displacement. However, the interleukin-6 level was less than 100 U/mL (range, 20 to 75 U/mL) in all patients with masticatory muscle disorder.	IL-6 associated with acute TMD
Fujita et al. (1999) [28]	P substance	Human disc and synovial fluid	Expression of substance P seems to be closely related to histopathological changes of the human TMJ with internal derangement.	Substance P associated with internal derangement
Suzuki et al. (1999) [29]	BMP-2	Human discs	BMP-2 was predominantly localized in chondrocytes around the damaged areas of the articular disks. BMP-2 expression was also found in synovial cells and endothelial cells of blood vessels.	BMP-2 associated with TMD
Yoshida et al. (1999) [30]	CD34 antigen	Human disc and synovial membrane	CD34 is suggested to be correlated with the process of angiogenesis induced by internal derangement of the TMJ.	Vascular endothelium and angiogenesis
Yoshida et al. (1999) [30]	TGF-beta and Tenascin	Human synovial membranes	TGF-beta and tenascin were distributed in the affected synovial membrane of TMJ with internal derangement. These findings suggested that TGF-beta and tenascin might have a close relationship with synovitis, followed by tissue repair.	TGF-beta and tenascin in TMD
Leonardi et al. (2000) [31]	CD44 Standard Form	Human discs	The up-regulation of CD44H observed in some dysfunctional TMJ discs seems to indicate a prevention of apoptosis in fibroblast-like cells and an important role in phenotypical change of fibrochondrocytes into chondroblastlike cells, enabling the aggregation of chondroid tissue pericellular matrix components.	Prevention of apoptosis in TMJ discs
Ogura et al. (2002) [25]	IL-1 Beta and IL-6	Human synovial cells	IL-1 beta increased IL-6 production in synovial cells. Enhanced production of IL-6, which is associated with bone resorption and inflammatory response, seems to be related to the progression of TMD.	IL-6 associated with TMD
Suzuki et al. (2002) [32]	IL-1 Beta and TNF-alpha	Human synovial fluids	IL-1beta and TNF-alpha may be involved with TMJ internal derangement and coordinately play a role in pathogenesis of TMJ internal derangement.	
Tobe et al. (2002) [33]	IL-1 Beta and IL-8	Human synovial cells	IL-1beta stimulated IL-8 production through an increase in IL-8 gene expression in HTS cells, which may be associated with the increase in infiltrating inflammatory cells seen in the synovial membrane of TMJ disorders.	IL-8 associated with TMD
Leonardi et al. (2003) [34]	VEGF	Human Disks	In disc specimens from internal derangement of the TMJ with significant tissue degeneration/regeneration, VEGF was consistently expressed.	VEGF expression in human disc with TMD
Sato et al. (2003) [26]	IL-6	Human synovial tissue	In synovial tissues from 21 of the 46 joints with internal derangement, interleukin 6 (IL-6) was expressed in the synovial lining cells and in the mononuclear cells infiltrating the periphery of the blood vessels.	IL-6 associated with TMD
Suzuki et al. (2003) [35]	Bradykinin	Human synovial tissues and fluids	Bradykinin was also detected in 19 patients’ TMJ synovial fluids and the average of bradykinin concentration in the synovial fluids of patients was higher than that of the healthy controls. Although a statistically significant correlation was not observed, these findings support the hypothesis that bradykinin may also be involved in the pathogenesis of TMJ pain and synovitis.	Bradykinin associated with pathogenesis of TMD pain and synovitis.
Sato et al. (2003) [36]	VEGF	Human synovial tissues	VEGF may have an important role in the genesis of joint effusion.	VEGF and joint effusion
Tanimoto et al. (2004) [37]	TGF-beta	Rabbit synovial membranes	TGF-beta 1 enhances the expression of HAS2 mRNA in the TMJ synovial membrane fibroblasts and may contribute to the production of high-molecular-weight HA in the joint fluid.	TGF-beta associated with high molecular weight hyaluronan and more viscous fluid
Kaneyama et al. (2005) [38]	Tumor Necrosis Factor-Alpha, interleukin-6, interleukin-1beta, Soluble Tumor Necrosis Factor Receptors I and II, interleukin-6 Soluble Receptor, interleukin-1 Soluble Receptor Type II, interleukin-1 Receptor Antagonist	Human synovial fluid	The concentrations of TNF-alpha, IL-6, IL-1beta, sTNFR-I, and sTNFR-II were significantly higher in the synovial fluid of patients than in controls (*p* < 0.05). TNF-alpha level was positively correlated with those of IL-6, sTNFR-I, and sTNFR-II. In particular, there was a highly significant positive correlation between sTNFR-I and sTNFR-II.	All associated with TMD
Yamaguchi et al. (2005) [39]	Hypoxia and interleukin-1beta and MMPs	Rabbit discs	The results showed that the combination of hypoxia and IL-1beta caused a significant increase in MMP-1, MMP-3, MMP-9 and MMP-13 mRNA.	Hypoxia and IL-1beta increases MMPs
Ogura et. Al. (2005) [40]	TNF-alpha	Human synovial membranes	Production of interleukin (IL)-8, growth-related oncogene (GRO)-alpha, monocyte chemoattractant protein (MCP)-1, and regulated upon activation normal T-cell expressed and secreted (RANTES) protein by synovial fibroblasts was increased by TNF-alpha.	Increased protein production of chemokines by synovial fibroblasts in response to TNF-alpha treatment.
Sato et. Al. (2007) [41]	IL-8	Human synovial tissues	IL-8 was up-regulated in inflamed synovial tissues in patients with internal derangement.	IL-8 associated with TMD with internal derangement
Matsumoto et al. (2006) [42]	Cytokines	Human synovial fluid	In synovial fluid samples, angiogenin (Ang), fibroblast growth factor (FGF)-9, insulin-like growth factor-binding protein (IGFBP)-3, interleukin (IL)-1alpha, IL-1beta, IL-8, inducible protein (IP)-10, macrophage inflammatory protein (MIP)-1beta, osteoprotegerin (OPG), transforming growth factor (TGF)-beta2, tissue inhibitor of metalloproteinase (TIMP)-1, TIMP-2, tumor necrosis factor (TNF)-beta and vascular endothelial growth factor (VEGF) were detectable. Furthermore, the expression levels of Ang, brain-derived neurotrophic factor (BDNF), FGF-4, FGF-9, IGFBP-2, IL-8, MIP-1beta, OPG, pulmonary and activation-regulated protein (PARC), TGF-beta2, TIMP-2 and VEGF were significantly associated with the presence of JE; among these, nine cytokines (Ang, BDNF, FGF-4, FGF-9, IGFBP-2, MIP-1beta, PARC, TGF-beta2 and TIMP-2) were hitherto not described in TMD.	Cytokine expression and TMD
Deschner et al. (2007) [43]	Insulin-like Growth Factor 1	Rat discs	Continuous biophysical strain seems to downregulate the expression of the IGF system and may reduce the potential of fibrocartilage for growth and repair.	IGF-1 associated with TMJ strain
Sato et al. (2007) [43]	IL-8	Human synovial tissue	IL-8 was up-regulated in inflamed synovial tissues in patients with internal derangement.	IL-8 associated with TMD
Ke et al. (2007) [44]	NF-kB and TNF-alpha	Human synovial fibroblasts	Activation of NF-kB is responsible for TNF-alpha-induced COX-2 expression in synovial fibroblasts from the TMJ.	NFkB, TNF-alpha, and COX-2 associated with TMD
Tojyo et al. (2008) [45]	Hypoxia and interleukin-1beta	Human synovial fluid and discs	The combination of hypoxia and interleukin-1beta caused a significant increase in tenascin-C protein and mRNA of synovial fibroblasts, but not in disc cells.	Hypoxia and interleukin-1beta increase tenascin-C protein
Leonardi (2011) [46]	TRAIL and DR5- and CASP3-dependent apoptosis	Human discs	Apoptosis involvement in the angiogenesis as a self-limiting process in patients with temporomandibular joint.	Apoptosis activation
Kaneyama (2010) [23]	Cytokine Receptors	Human synovial fluid	Mean concentrations of cytokine receptors (tumor necrosis factor receptors I and II, interleukin (IL) 6 soluble receptor, IL-1 soluble receptor type II, and IL-1 receptor antagonist and protein)in the synovial fluid were significantly higher in the 30 joints with JE than in the 25 joints without JE.	Increase in cytokine receptor levels with TMD
Leonardi et al. (2011) [47]	TNF-Related Apoptosis-Inducing Ligand Expression	Human discs	Cell loss due to the involvement of TRAIL apoptotic pathway seems, in part, responsible for TMJ disk degeneration.	Apoptosis and TMD
Kim (2012) [48]	Granulocyte Macrophage Colony stimulating Factor (GM-CSF), interferon (INF), interleukin (IL)-1β, IL-2, IL-4, IL-5, IL-6, IL-8, IL-10 and tumor necrosis factor (TNF)-α	Human synovial fluid	Granulocyte Macrophage Colony stimulating Factor (GM-CSF), interferon (INF), interleukin (IL)-1β, IL-2, IL-6, IL-8, IL-10 and tumor necrosis factor (TNF)-α were detected in the TMD group, whereas no cytokines were detected in the control group. IL-4 and IL-5 were not detected in either the TMD group or in the control group.	Certain cytokines (noted left) associated with TMD
Camejo (2013) [49]	Fas Ligand (FasL)	Human discs	A higher area of in situ immunostaining of FasL was found in temporomandibular discs with ADDwR ^1^.	Apoptosis and TMD
Sicurezza (2013) [50]	β-Defensin	Human discs	The presence of β-defensin-4 in human TMJ discs affected by ADDwoR ^2^ was found, hypothesizing its possible role in articular bone disruption.	β-Defensin and disc degeneration
Caporal et al. (2017) [29]	IL-6	Human discs	No significant differences were observed between the groups ADDwR and ADDwoR, and with and without OA, in respect to the expression of IL-6.	No difference in IL-6 between two TMD groups
Nakagawa et al. (2017) [49]	IFN-γ and TNF-α	Human synovial fluid	TNF-α and IFN-γ function in a cooperative manner to regulate inflammatory chemokine expression in synovial fibroblasts.	TNF-α and IFN-γ associated with inflammatory regulation in TMJ.
Kaya et al. (2018) [50]	Chemerin	Human synovial fluid	Chemerin in synovial fluid may play a role as a predisposing factor and may represent a novel potential prognostic biochemical marker in the pathogenesis of TMJ disorders.	Chemerin associated with TMD
Sorenson et al. (2018) [51]	IL-1	Human synovial fluid	Articles that compared IL-1 concentrations in TMD vs. control groups found significant differences.	IL-1 associated with TMD
Luo et al. (2019) [52]	IL-37b	Human synovial fluid	IL-37b suppressed inflammation and inhibited osteoclast formation.	Anti-inflammatory effects of IL-37.

^1^ Anterior Disc Displacement with Reduction; ^2^ Anterior Disc Displacement without Reduction.

**Table 3:** Proteoglycans (Tenascin, vimentin, fibronectin, decorin, biglycan, versican, elastin).

Authors	IHC Marker	Study Design and Tissue Expression	Results	Conclusions
Yoshida et al. (1996) [56]	Tenascin	Human synovial membranes	Synovial cells in the synovial membrane produce tenascin in the diseased human temporomandibular joint	Tenascin associated with TMD
Yoshida et al. (1997) [28]	Tenascin	Human disc and synovial membranes	Tenascin is expressed specifically in the portion of the TMJ synovial membrane affected with internal derangement	Tenascin associated with degenerated tissue
Mizoguchi et al. (1998) [5]	Biglycan, Decorin and Large Chondroitin-Sulphate Proteoglycan	Rat discs	Staining for biglycan was intense in the posterior band. In contrast, staining for decorin was faint in the intermediate zone and the central part of the posterior band, moderate in the anterior and posterior attachments and most intense in the junction between the anterior band and attachment. Similarly, there was intense staining for large chondroitin-sulphate proteoglycan in the peripheral band.	Presence of biglycan, decorin and large chondroitin-sulphate proteoglycan in disc.
Kuwabara et al. (2002) [57]	Decorin and Biglycan	Rat discs	Regional differences in staining for decorin became prominent at 4, 8 and 16 weeks; decorin was more abundant in the peripheral area of the band than in the central area. In contrast, staining for biglycan was evenly distributed throughout the disc until 4 weeks, and after that became intense in the anterior and posterior bands.	Decorin and biglycan present in discs
Leonardi et al. (2002) [58]	Vimentin and Alpha-Smooth Muscle Actin	Human discs	Vimentin is expressed by all disc cell populations and it does not appear to be influenced by any disease condition of the disc; on the other hand the up-regulation of alpha-SM actin immunolabelling seems to be correlated with histopathological findings of tears and clefts.	Localization of Vimentin and Alpha-Smooth Muscle Actin in TMJ discs
Yoshida et al. (2002) [59]	Tenascin	Human synovial fluid and discs	Tenascin was produced specifically in synovial cells and vascular endothelial cells and. Fibroblasts were affected in the portion of TMJ with internal derangement.	Tenascin expression in TMD
Kondoh et al. (2003) [60]	Type II Collagen	Human discs	The percentage of type II collagen in immunoreactive disc cells was significantly higher in the outer part (the articular surfaces) than in the inner part (the deep central areas) of the disc.	Increased collagen synthesis in TMJ discs
Yoshida et al. (2004) [54]	Vimentin	Human discs and synovial membrane	There was an obvious distinction of vimentin immunoreactivity between the control specimens and internal derangement cases, in the posterior and/or anterior loose connective tissues. In particular, intensive vimentin expression was detected in the hypertrophic synovial membrane of internal derangement cases.	Vimentin present in synovial membrane of TMD
Leonardi et al. (2004) [61]	Fibronectin	Human discs	The findings suggest that TMJ disc tissue can express fibronectin and that the expression is more pronounced in disc specimens of patients with internal derangements.	Fibronectin associated with internal derangements
Paegle et al. (2005) [62]	Proteoglycans aggrecan, versican, biglycan, decorin, fibromodulin and hyaluronan synthase 1	Human discs	Aggrecan expression was higher in patients with chronic closed lock. Within posterior disc attachment specimens, chronic closed lock showed a tendency for higher expression of biglycan and hyaluronan synthase 1.	Proteoglycans associated with chronic closed lock
Toriya et al. (2006) [55]	Versican-core protein of a large chondroitin sulphate proteoglycan	Rat discs	Growth-related changes and regional differences exist in the expression of versican in the TMJ discs of growing rats.	Versican associated with TMD
Moraes et al. (2008) [63]	Collagen Type IV	Human fetus discs	Marker of type IV collagen showed the presence of blood vessels in the central region of the temporomandibular disc.	Collagen present in disc formation
Li et al. (2008) [64]	Hyaluronan and hyaluronan synthase	Human synovial membrane	IL-1beta functions on regulating HAS expression and consequently promoting the secretion of HA in synovial lining cells from TMJ.	Hyaluronan present in synovial membranes
Natiella et al. (2009) [65]	Collagen Type I and Fibronectin	Human synovial fluid and discs	Disc specimens with advanced morphologic pathology showed significant labeling for fibronectin in 3 of 3 cases and for collagen I in 4 of 4 cases. There was no considerable difference in detection of either fibronectin or collagen I in TMJ synovial aspirates from patients with advanced disc pathology compared with controls.	Fibronectin found in pathologic TMJ discs
Matsumoto et al. (2010) [66]	Hyaluronan and hyaluronan synthase (HAS)	Human discs	Hyaluronan synthase-3 is related to the pathological changes of human TMJ discs affected by ID.	HAS associated with TMJ degradation
Kiga et al. (2010) [67]	Lumican, CD34 and vascular endothelial growth factor	Human discs	Assembly and regulation of collagen fibers. Lumican new collagen network by fibroblast-like cells. Presence of VEGF and CD34 inside the deformed disc.	Lumican expression associated with increased CD34 and VEGF in TMJ
Fang et al. (2010) [68]	Chondromodulin-1 (ChM-1)	Rabbit discs	ChM-1 may play a role in the regulation of TMJ remodeling by preventing blood vessel invasion of the cartilage.	Anti-angiogenic factor in TMJ discs
Kiga (2011) [69]	Lumican and fibromodulin under interleukin-1 beta (IL-1 β)-stimulated conditions	Human discs	Lumican and fibromodulin display different behaviors and that lumican may promote regeneration of the TMJ after degeneration and deformation induced by IL-1 β.	IL-1 β induces a significant increase in lumican mRNA, but not in fibromodulin mRNA.
Leonardi et al. (2011) [70]	Lubricin	Human discs	A longstanding TMJ disc injury, affects lubricin expression in the TMJ disc tissue and not its surfaces; moreover, lubricin immunostaining is not correlated to TMJ disc histopathological changes.	No correlation between TMD and lubricin expression
Leonardi et al. (2012) [71]	Lubricin	Human discs	Lubricin may have a role in normal disc posterior attachment physiology through the prevention of cellular adhesion as well as providing lubrication during normal bilaminar zone function.	Lubricin associated with TMJ homeostasis
Hill et al. (2014) [72]	Lubricin	Rat synovial fluid	Lack of lubricin in the TMJ causes osteoarthritis-like degeneration that affects the articular cartilage as well as the integrity of multiple joint tissues.	Protective effects of lubricin on TMJ
Shinohara et al. (2014) [73]	Tenascin-C (TNC)	Mice synovial fluid and disc	TNC was expressed in the wounded TMJ disc and mandibular fossa, lack of TNC may reduce fibrous adhesion formation in the TMJ.	TNC associated with TMD and fibrous adhesion formation
Leonardi (2016) [74]	Lubricin	Human synovial fluid	Lubricin levels were inversely correlated with age and to Wilkes score. Lubricin decreases in synovial fluid with advanced disease.	Protective effects of lubricin on TMJ

**Table 4:** Hormones (estrogen, progesterone, relaxin).

Authors	IHC Marker	Study Design and Tissue Expression	Results	Conclusions
Naqvi et al. (2004) [76]	Relaxin and β-estradiol	Rabbit discs	Relaxin and β-estradiol plus relaxin induced the MMPs collagenase-1 and stromelysin-1 in fibrocartilaginous explants, accompanied by a loss of GAGs and collagen but not altering the synthesis of GAGs.	Relaxin associated with degenerative effects on TMJ
Hashem et al. (2006) [77]	Relaxin, β-estradiol, and progesterone alone or in various combinations.	Rabbit discs	Collagen caused by β-estradiol, relaxin, or β-estradiol + relaxin causes loss of disc glycosaminoglycans, Progesterone prevented relaxin- or β-estradiol-mediated loss of these molecules.	Estradiol and relaxin causes loss of glycosaminoglycans, and progesterone prevents it.
Wang et al. (2008) [78]	Estrogen receptors α, β, relaxin receptors LGR7 and LGR8, and progesterone receptor	Mice discs	TMJ cells had higher ER-α (>2.8-fold), ER-β (>2.2-fold), LGR7 (>3-fold) and PR (>1.8-fold), and lower LGR8 (0.5-fold).	Estrogen, relaxin, and progesterone within TMJ discs
Kapila et al. (2009) [11]	β-estradiol or relaxin and progesterone/estrogen receptors (ER)-α and –β, relaxin-1 receptor (RXFP1, LGR7), and INSL3 receptor (RXFP2, LGR8)	Rabbit discs	Relaxin produces a dose-dependent induction of tissue-degrading enzymes of the matrix metalloproteinase family, specifically MMP-1, MMP-3, MMP-9, and MMP-13 in cell isolates and tissue explants from TMJ fibrocartilage. The induction of these MMPs is accompanied by loss of collagen and glycosaminoglycans, which was blocked by a pan-MMP inhibitor. Progesterone attenuated the induction of MMPs.	Estrogen, relaxin, and progesterone within TMJ discs
Puri et al. (2009) [75]	Estrogen receptor α	Rat disc and synovial fluid	The number of ER α -positive cells in the TMJ was not affected by inflammation or 17 beta-estradiol with exception of the retrodiscal tissue	Low estrogen receptor in normal TMJ
McDaniel et al. (2014) [79]	Estrogen and progesterone	Baboon discs	Treatment of baboon TMJ disc cells with estrogen led to reduced PRG4 promoter activity and mRNA expression in vitro.	Negative regulation of PRG4 by estrogen.
Park et al. (2019) [80]	Estrogen and progesterone	Mouse discs	Administration of Estrogen but not Progesterone caused a significant loss of TMJ collagen and glycosaminoglycans, accompanied by amplification of ERα and specific increases in MMP9 and MMP13 expression.	E2-mediated upregulation of MMP9 and MMP13.

**Table 5:** Miscellaneous (aquaporin, DKK1, etc).

Authors	IHC Marker	Study Design and Tissue Expression	Results	Conclusions
Leonardi et al. (2002) [87]	Heat shock protein 27	Human discs	HSP-27 upregulates in internal derangement specimens with major histopathological changes; it is not expressed or only weakly expressed in TMJ discs of fetuses and normal TMJ discs.	HSP-27 upregulation in TMD
Huang et al. (2004) [82]	Bcl-2 and Bax	Rabbit discs	Chondrocyte cytoplasm in the disc exhibited a high intensity for Bcl-2, while Bax activity was only sporadically observed. Bcl-2 and Bax proteins are present in TMJ cartilage and their expression patterns suggest that these oncoproteins are involved in chondrocyte survival or death via apoptotic pathways.	Apoptotic pathways within TMJ discs
Fujimura et al. (2006) [84]	Synovial fluid proteins	Human synovial fluid	Approximately 22 different protein bands with molecular weights ranging from 14 to 700 kd were clearly discernible on electrophoresis. The relative amounts of specific proteins in the SF of the TMD group were also different from those in the AS group (*p* < 0.05). The major difference in total protein concentration appeared to be due to the increased abundance of relatively high molecular weight proteins (>140 kd) in the TMD patients as compared to the AS group.	Increased concentration of synovial proteins associated with increased stage of TMD.
Loreto (2012) [85]	Aquaporin	Human discs	AQP1 is normally expressed in the TMJ disc and confirm a role for it in the maintenance of TMJ homeostasis.	Aquaporin acts on TMJ homeostasis
Loreto (2012) [85]	Aquaporin	Human discs	Aquaporin-1 is expressed and upregulated in temporomandibular joint with anterior disc displacement (both with and without reduction).	Channel protein involved in plasma membrane water permeability
Jiang (2015) [81]	Dickkopf-related Protein 1 (DKK-1)	Human synovial fibroblasts	DKK-1 is associated with angiogenesis in the synovial fluid of patients with TMD.	DKK-1 associated with TMD
Kobayashi (2017) [53]	Elastin-derived peptides	Human synovial fluid	Upregulation of IL-6 and MMP-12 expression by EDPs may be mediated through elastin-binding proteins (EBP) and a protein kinase A signaling cascade.	Elastin-derived peptides modulate the inflammatory cascade
Castorina et al. (2019) [83]	P53 and VEGF	Human discs	P53 and VEGF expression in TMJ discs with internal derangement correlate with degeneration.	P53 and VEGF associated with TMD

## Data Availability

Data can be found on PubMed.
